# Adaptive preconditioning in neurological diseases – therapeutic insights from proteostatic perturbations

**DOI:** 10.1016/j.brainres.2016.02.033

**Published:** 2016-10-01

**Authors:** B. Mollereau, N.M. Rzechorzek, B.D. Roussel, M. Sedru, D.M. Van den Brink, B. Bailly-Maitre, F. Palladino, D.B. Medinas, P.M. Domingos, S. Hunot, S. Chandran, S. Birman, T. Baron, D. Vivien, C.B. Duarte, H.D. Ryoo, H. Steller, F. Urano, E. Chevet, G. Kroemer, A. Ciechanover, E.J. Calabrese, R.J. Kaufman, C. Hetz

**Affiliations:** a Univ Lyon, ENS de Lyon, Univ Claude Bernard, CNRS UMR5239, INSERM U1210, Laboratory of Biology and Modelling of the Cell, F-69007, Lyon, France; b Centre for Clinical Brain Sciences, Chancellor’s Building, University of Edinburgh, Edinburgh EH16 4SB, United Kingdom; c Royal (Dick) School of Veterinary Studies, University of Edinburgh, Easter Bush Campus, Roslin, Midlothian EH25 9RG, United Kingdom; d Inserm, UMR-S U919 Serine Proteases and Pathophysiology of the Neurovascular Unit, 14000 Caen, France; e INSERM U1065, C3M, Team 8 (Hepatic Complications in Obesity), Nice, France; f Biomedical Neuroscience Institute, Faculty of Medicine, University of Chile, Santiago, Chile; g Center for Molecular Studies of the Cell, Program of Cellular and Molecular Biology, Institute of Biomedical Sciences, University of Chile, Santiago, Chile; h Center for Geroscience, Brain Health and Metabolism, Faculty of Medicine, University of Chile, Santiago, Chile; i Department of Immunology and Infectious Diseases, Harvard School of Public Health, Boston, MA, USA; j ITQB-UNL, Av. da Republica, EAN, 2780-157 Oeiras, Portugal; k Inserm, U 1127, F-75013 Paris, France; l CNRS, UMR 7225, F-75013 Paris, France; m Sorbonne Universités, UPMC Univ Paris 06, UMR S 1127, F-75013 Paris, France; n Institut du Cerveau et de la Moelle épinière, ICM, F-75013 Paris, France; o Genes Circuits Rhythms and Neuropathology, Brain Plasticity Unit, CNRS UMR 8249, ESPCI ParisTech, PSL Research University, 75005 Paris, France; p ANSES, French Agency for Food, Environmental and Occupational Health & Safety, Neurodegenerative Diseases Unit, 31, avenue Tony Garnier, 69364 Lyon Cedex 07, France; q CNC-Center for Neuroscience and Cell Biology, University of Coimbra, Faculty of Medicine, Rua Larga, and Department of Life Sciences, University of Coimbra, 3004-504 Coimbra, Portugal; r Department of Cell Biology, New York University School of Medicine, New York, NY, USA; s Howard Medical Institute, The Rockefeller University, 1230 York Avenue, New York, NY 10021, USA; t Washington University School of Medicine, Department of Internal Medicine, St. Louis, MO 63110 USA; u Inserm ERL440 “Oncogenesis, Stress, Signaling”, Université de Rennes 1, Rennes, France; v Centre de Lutte Contre le Cancer Eugène Marquis, Rennes, France; w Equipe 11 labellisée par la Ligue contre le Cancer, Centre de Recherche des Cordeliers, Paris, France; x Cell Biology and Metabolomics platforms, Gustave Roussy Comprehensive Cancer Center, Villejuif, France; y INSERM, U1138, Paris, France; z Université Paris Descartes, Sorbonne Paris Cité, Paris, France; aa Université Pierre et Marie Curie, Paris, France; ab Pôle de Biologie, Hôpital Européen Georges Pompidou, AP-HP, Paris, France; ac Karolinska Institute, Department of Women׳s and Children׳s Health, Karolinska University Hospital, Stockholm, Sweden; ad The Polak Cancer and Vascular Biology Research Center, The Rappaport Faculty of Medicine and Research Institute, Technion-Israel Institute of Technology, Haifa 30196, Israel; ae Department of Environmental Health Sciences, University of Massachusetts, Morrill I, N344, Amherst, MA 01003, USA; af Degenerative Diseases Program, Sanford Burnham Prebys Medical Discovery Institute, 10901 N. Torrey Pines Rd., La Jolla, CA 92037, USA

**Keywords:** ARE, anti-oxidant response element, ASK1, apoptosis signal regulating kinase 1, ATF4, activating transcription factor 4, ATF6α, activating transcription factor 6α, BI-1, Bax-inhibitor-1, Bim, Bcl-2-interacting mediator of cell death, BiP/GRP78, binding immunoglobulin protein, BDNF, brain-derived neurotrophic factor, CDNF, cerebral dopamine neurotrophic factor, CIRBP, cold-inducible RNA binding protein, CHOP, C/EBP-homologous protein, DA, dopaminergic, EGFR, epidermal growth factor receptor, ER, endoplasmic reticulum, ERAD, ER-associated protein degradation, eIF2α, eukaryotic translation initiation factor 2α, GADD34, growth arrest and DNA damage–inducible 34, GEF, guanine nucleotide exchange factor, HD, Huntington’s disease, Hsp70, heat shock protein 70, HO-1, heme oxygenase-1, HIF-1, hypoxia-inducible transcription factor-1, iPSC, induced pluripotent stem cell, IRE1, inositol-requiring enzyme 1, IRI, ischemia reperfusion injury, JNK, Jun amino terminal kinase, LRRK2, leucine-rich repeat kinase 2, MANF, mesencephalic astrocyte-derived neurotrophic factor, MCAO, middle cerebral artery occlusion, MPTP, 1-Methyl-4-phenyl-1,2,3,6-tetrahydropyridine, nAChRs, nicotinic acetylcholine receptors, NF-KB, nuclear factor-kappa B, OGD, oxygen and glucose deprivation, PD, Parkinson’s disease, PHD, prolyl-hydroxylases, PERK, protein kinase RNA-like ER kinase, PMD, protein misfolding disorder, PUMA, p53 upregulated modulator of apoptosis, RIDD, regulated IRE1-dependent decay, RBM3, RNA binding motif 3, ROS, reactive oxygen species, SERCA, sarcoplasmic-ER Mg2+/Ca2+ ATPase, SOD, superoxide dismutase, SNpc, *substantia nigra pars compacta*, S1P, site 1 protease, S2P, site 2 protease, tPA, tissue plasminogen activator, Ub, ubiquitin, UPR, unfolded protein response, UPS, ubiquitin proteasome system, XBP1, x-box binding protein 1, xCT, cystine/glutamate antiporter, Wfs1, wolframin, 6-OHDA, 6-hydroxydopamine, 3-MA, 3-methyladenine, ER stress, Proteasome, Parkinson׳s disease, Ischemia, Hormesis, Autophagy, Wolfram syndrome, Glioblastoma

## Abstract

In neurological disorders, both acute and chronic neural stress can disrupt cellular proteostasis, resulting in the generation of pathological protein. However in most cases, neurons adapt to these proteostatic perturbations by activating a range of cellular protective and repair responses, thus maintaining cell function. These interconnected adaptive mechanisms comprise a ‘proteostasis network’ and include the unfolded protein response, the ubiquitin proteasome system and autophagy. Interestingly, several recent studies have shown that these adaptive responses can be stimulated by preconditioning treatments, which confer resistance to a subsequent toxic challenge – the phenomenon known as hormesis. In this review we discuss the impact of adaptive stress responses stimulated in diverse human neuropathologies including Parkinson׳s disease, Wolfram syndrome, brain ischemia, and brain cancer. Further, we examine how these responses and the molecular pathways they recruit might be exploited for therapeutic gain.

*This article is part of a Special Issue entitled SI:ER stress*.

## Highlights

• The proteostasis network is perturbed in neurological diseases.
• UPR, UPS and autophagy are adaptive responses to maintain cell function.
• Adaptive responses induced by preconditioning could be used for therapeutics.

## 1 Introduction

The long-term health of cells critically relies on protein quality control, since damaged, misfolded or aggregated proteins cause proteotoxic stress that impairs cell function (Glickman and Ciechanover, 2002, Morimoto, 2008, Goldberg, 2003, Ihara et al., 2012). The healthy brain adapts to cellular stresses that disrupt protein homeostasis (referred to as proteostasis) (Balch et al., 2008). Proteostatic perturbations are induced by a variety of stressors including (but not limited to) pathological protein inhibition of protein clearance pathways and oxygen deprivation. Depending on the type and intensity of the stress, distinct but interconnected adaptive responses are set in motion; these include autophagy, the unfolded protein response (UPR) (Box 1), the ubiquitin proteasome system (UPS) (Box 2), the anti-oxidant response, and heat- and cold-shock responses (Martins et al., 2011, Mollereau et al., 2014, Mollereau, 2015). A subtoxic level of stress engages these adaptive responses and elicits preconditioning, thus conferring protection against further toxic insults (Mattson, 2008, Rzechorzek et al., 2015). This phenomenon, generally referred to as ‘hormesis’, is strongly conserved in evolution and can be induced by many and varied perturbations including (among others) oxidative stress, ER stress, inflammatory stimuli, and temperature shift, all of which disrupt cellular proteostasis (Rutkowski et al., 2006, Calabrese, 2014, Mollereau et al., 2014, Rzechorzek et al., 2015). Of note, a recent review compiled the effect of 154 distinct conditioning agents used in preconditioning or postconditioning experiments (Calabrese, 2016a). In the last few years an increasing number of studies have focused on the importance of hormesis in pathologies such as neurodegenerative disease, cancer, diabetes and aging (Hetz and Mollereau, 2014, Martins et al., 2011, Mollereau, 2013, Mollereau et al., 2014, Perri et al., 2015). In this review, we focus on Parkinson׳s disease (PD), Wolfram syndrome, brain ischemia, and brain cancer (glioma) for which perturbations of cellular proteostasis (and in some cases adaptations to these) have been clearly established. We also discuss the therapeutic potential of engaging or interfering with hormetic responses in a context-dependent manner.

## 2 Stress adaptation in Parkinson׳s disease

PD is a movement disorder characterized by the selective loss of dopaminergic (DA) neurons in the *substantia nigra pars compacta* (SNpc) resulting in motor symptoms such as bradykinesia, rigidity and resting tremor (Hirsch et al., 2013). DA neuron loss involves severe proteostatic alterations, evidenced by the accumulation of Lewy bodies - cytoplasmic protein inclusions enriched in α-synuclein. A subset of patients with early-onset familial PD carry mutations or duplications in the gene coding for α-synuclein, which have been linked to misfolding and aggregation of the protein (Conway et al., 1998). Proteostatic perturbation, misfolding and subsequent aggregation of α-synuclein are believed to play major roles in the pathomechanism of PD, which belongs to a family of neurological conditions known as protein misfolding disorders (PMD) (Hetz and Mollereau, 2014, Soto, 2003). Here we highlight the tight association between genes that are frequently mutated in familial PD and alterations in the UPR machinery. We will also discuss the potential value of modulating ER-hormesis as a neuroprotective mechanism in PD.

### 2.1 Endogenous adaptive response induced in PD

The UPR is an adaptive response that is activated to cope with ER stress (*Box 1*). In PD, ER stress components constitute early biomarkers; for example phosphorylated PERK is found in DA neurons of the SNpc and colocalizes with α-synuclein (Hoozemans et al., 2007, Hoozemans et al., 2012). Induction of most UPR signaling responses is reported in different toxicological models of PD *in vitro* and *in vivo* (Bauereis et al., 2011, Mercado et al., 2013). In addition, the generation of neurons from induced pluripotent stem cell (iPSC) lines derived from PD patients carrying α-synuclein mutations revealed a major dysfunction in proteostasis (Chung et al., 2013). This study showed that ER stress and UPR activation are prominent features in PD-associated cell alterations. Several studies also report that an impairment in vesicular trafficking is commonly observed in PD, which may be responsible for UPR activation and perturbation of proteostasis (Cooper et al., 2006, Gitler et al., 2008) and is reviewed in (Mercado et al., 2015).

**Fig. 1:**
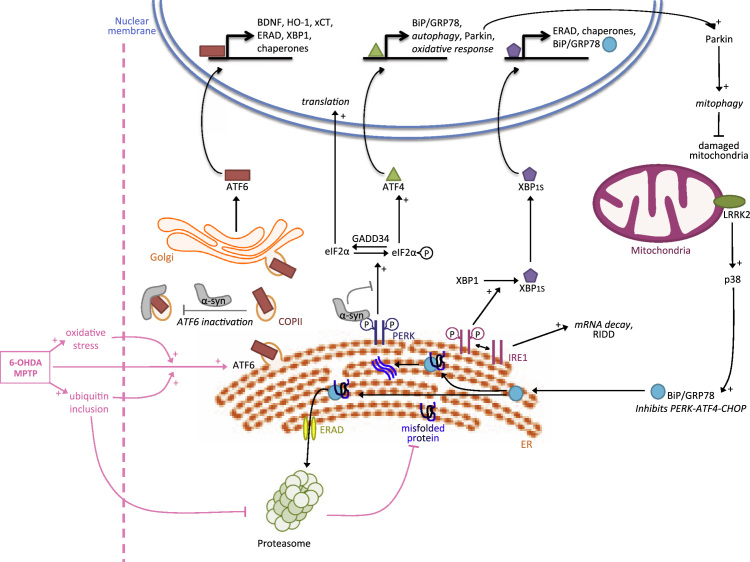
The adaptive UPR in Parkinson׳s disease. In PD the UPR is activated. MPTP or 6-OHDA treatments induce ATF6 activation, oxidative stress and ubiquitin inclusions, which inhibit the proteasome. ATF6 confers neuroprotection to DA neurons by promoting ERAD factors that target misfolded protein to the proteasome and XBP1, which further alleviates ER stress by inducing expression of chaperones. ATF6 also induces the expression of BDNF, HO-1 and xCT that protects DA neurons. ATF6-mediated protection is hampered by α-synuclein which interacts with ATF6, inhibiting its activation by interfering with trafficking of COPII vesicles. The PERK/ATF4 branch is also activated and contributes to neuroprotection by inducing the expression of Parkin which in turn promotes mitophagy. α-synuclein also associates with PERK and may interfere with its function. IRE1/XBP1 contributes to neuroprotection by inducing BiP expression which limits overactivation of PERK and proapoptotic CHOP expression. BiP can also be induced via LRRK2 and the p38 pathway.

In the last few years it has become clear that the UPR is a double-edged sword in PD: it is cytoprotective when activated at moderate levels during the early course of the disease, but cytotoxic when activated in an intense and sustained manner in the late phase of the disease (Hetz and Mollereau, 2014, Silva et al., 2005). Here we outline the adaptive arms of the UPR that involve several protective pathways, allowing neurons to cope for many years with cellular stress in PD (Fig. 1).

Several studies have described an important contribution of the α subtype of ATF6 (ATF6α) to neuroprotection in mouse models of PD. Mice injected intraperitoneally with the DA neurotoxin 1-methyl-4-phenyl-1,2,3,6-tetrahydropyridine (MPTP) - which induces oxidative stress, ubiquitin inclusions and a selective loss of DA neurons - exhibited an activation of ATF6α and ERAD in DA neurons. Importantly, mice deficient for ATF6α were more sensitive to MPTP compared to wild type animals indicating that ATF6α confers neuroprotection (Egawa et al., 2011). Interestingly, another study proposed that neuroprotection is mediated at least in part by ATF6α in the astrocytes of mice submitted to MPTP treatment suggesting that UPR-associated neuroprotection may proceed through non-cell autonomous mechanisms (Hashida et al., 2012). The authors showed that MPTP induces activation of ATF6α in astrocytes, which in turn triggers the production of brain-derived neurotrophic factor (BDNF) and anti-oxidative genes, such as heme oxygenase-1 (HO-1) and the cystine/glutamate antiporter (xCT), conferring protection against DA neuron loss. Other essential neurotrophic factors including cerebral dopamine neurotrophic factor (CDNF) and mesencephalic astrocyte-derived neurotrophic factor (MANF) confer protection to DA neurons whilst interacting with UPR function (Voutilainen et al., 2015). Together these studies indicate that ATF6α is an important player in the adaptive response of DA neurons. A recent study showed that the ATF6α protective arm is inhibited by α-synuclein during the course of the disease (Credle et al., 2015). Specifically, α-synuclein interacted with ATF6α and inhibited the ER-Golgi transit of COPII vesicles that is required for ATF6α activation. This resulted in impaired ATF6α activation, reduced ERAD activity and increased apoptosis of DA neurons. The dysfunction of the ERAD machinery leads to the accumulation of ERAD substrates which is commonly observed in several neurodegenerative diseases including Alzheimer’s disease (AD), amyotrophic lateral sclerosis (ALS) and PD (Abisambra et al., 2013, Chung et al., 2013, Nishitoh et al., 2008). Thus although α-synuclein can primarily induce ER stress (Gorbatyuk et al., 2012), its specific interaction with ATF6α leads to the impairment of UPR adaptive function and ultimately contributes to disease pathogenesis, possibly by exacerbating protein misfolding.

The PERK/eIF2α/ATF4 branch, which is associated with activation of the transcription factor CHOP (a potentially toxic output of the UPR) also contributes to neuroprotection in PD (Bouman et al., 2011, Sun et al., 2013). For example, pharmacological enhancement of eIF2α phosphorylation with salubrinal has neuroprotective effects in PD models *in vivo* (Colla et al., 2012). It was also shown that Parkin, mutated in an autosomal recessive form of early-onset PD, is induced by ATF4 upon mitochondrial or ER stress to promote neuroprotection (Bouman et al., 2011). The loss of ATF4 in neuronal PC12 cells treated with MPTP or 6-hydroxydopamine (6-OHDA) resulted in decreased Parkin expression and enhanced death (Sun et al., 2013). Notably Parkin, which is also induced by the mitochondrial UPR, eliminates damaged mitochondria by activating mitophagy (Jin and Youle, 2013, Narendra et al., 2008). Interestingly, it was proposed that Parkin-mediated cell protection does not require its ubiquitin-ligase activity suggesting that it acts independently of the proteasome (Bouman et al., 2011).

Activation of IRE1/XBP1 also induces an efficient adaptive response in PD; it leads to the expression of chaperones such as BiP/GRP78 and ERAD factors that contribute to neuroprotection. We have reported that the developmental ablation of XBP1 in the nervous system protects DA neurons against a PD-inducing neurotoxin through an ER-hormesis compensatory mechanism (Valdes et al., 2014). Neuronal survival was mediated in part by preconditioning that resulted from the induction of an adaptive ER stress response. Furthermore, the consequences of manipulating the UPR network in PD has been tested using gene therapy (Castillo et al., 2015). We recently employed gene therapy to deliver active XBP1s into the SNpc, a strategy that provided neuroprotection and reduced striatal denervation in PD models (Valdes et al., 2014). Similarly, in mice treated with MPTP, adenoviral expression of XBP1s prompted survival of DA neurons. Neuroprotection was also observed by overexpression of BiP/GRP78 in rats expressing human α-synuclein (Gorbatyuk et al., 2012). BiP/GRP78 expression resulted in the downregulation of PERK and CHOP. Furthermore, in a study performed in *C. elegans* and human neuroblastoma cells, it was shown that BiP/GRP78 expression depends on the leucine-rich repeat kinase 2 (LRRK2), the most commonly mutated protein in PD. Induction of BiP/GRP78 resulted in neuroprotection against 6-OHDA treatment or α-synuclein expression possibly through activation of the p38 pathway (Yuan et al., 2011). Moreover, a mutated form of LRRK2 (G2019S) resulted in chronic activation of p38 in murine neurons and age-related DA-specific neurodegeneration in nematodes. Together these studies highlight the strong link between LRRK2 and the UPR, as well as the important roles of IRE1/XPB1 and Bip/GRP78 in the adaptive response in PD.

### 2.2 Adaptive responses induced by preconditioning in PD

In the last few years, *Drosophila* has become an important model to study the contribution of ER stress and mitochondrial dysfunction to cell death and neurodegeneration (Coulom and Birman, 2004, Mollereau, 2009, Rasheva and Domingos, 2009, Ryoo and Steller, 2007, Ryoo, 2015). Taking advantage of the crystal-like array that is formed by the 800 ommatidia of the adult *Drosophila* eye, which allows a fine visualization of degeneration, homologs of the UPR have been characterized in models of retinitis pigmentosa and PD (Kang and Ryoo, 2009, Lessing and Bonini, 2009, Mollereau and Domingos, 2005, Ryoo et al., 2007). In several studies, pharmacological or genetic manipulation of the UPR prompted preconditioning, whereby the adaptive arms of the UPR were activated offering protection from neurodegeneration in models of PD (Mollereau et al., 2014, Tsujii et al., 2015). The idea that ER-preconditioning/ER-hormesis may protect against neurodegeneration followed from the observation that *Drosophila* mutant in the ER-resident chaperone NinaA exhibited UPR activation and resistance to various apoptotic stimuli (Mendes et al., 2009). This result prompted the analysis of preconditioning of the ER in several animal and cellular models of PD. Preconditioning of the ER induced via injection of the ER stressor tunicamycin (inhibitor of N-glycosylation) reduced DA neuron loss and improved locomotor activity after stereotaxic injection of 6-OHDA in mice. Similar protection by tunicamycin administration was observed in a human neuroblastoma cell line treated with 6-OHDA and in *Drosophila* expressing human α-synuclein (Fouillet et al., 2012). Interestingly, UPR-mediated protection required XBP-1 and was associated with an increase in protective autophagy. This indicates an important protective role of the IRE1/XBP1 pathway and autophagy in preconditioning of the ER in models of PD.

As mentioned, specific ablation of XBP1 in the nervous system has been associated with an adaptive ER stress response in models of PD but also in models of ALS and HD (Hetz et al., 2009, Matus et al., 2009, Valdes et al., 2014, Vidal et al., 2012). In the ALS model, XBP1 ablation resulted in upregulated autophagy that protected mutant SOD1 transgenic mice against disease by eliminating mutant SOD1 aggregates (Hetz et al., 2009). Virtually identical observations were observed in HD models (Vidal et al., 2012). The neuroprotective effects of targeting XBP1 in the SNpc were correlated with the upregulation of several ER chaperones and autophagy markers (Valdes et al., 2014). Together these results indicate that XBP1 is an important switch in the control of the ER adaptive response.

ER-preconditioning also induces an anti-oxidant response that contributes to neuroprotection (Hara et al., 2011, Mendes et al., 2009). It was observed that pretreatment with thapsigargin (an inhibitor of the sarcoplasmic/ER Mg^2+^/Ca^2+^ ATPase; SERCA) conferred resistance in SH-SY5Y neuroblastoma cells challenged with 6-OHDA by upregulating HO-1. Thapsigargin stimulated the anti-oxidant response element (ARE) upstream of HO-1 (Hara et al., 2011). Interestingly, inhibition of store-operated calcium entry (SOCE) has also been shown to be protective against MPTP in PC12 cells. The pharmacological inhibition of SOCE, which resulted in inhibition of the release of intracellular Ca^2+^ from the ER, led to the expression of Homer1a, a scaffold protein with an anti-oxidant potential (Li et al., 2013).

A requirement for future therapy will be to promote a long lasting ER-adaptive response by fine-tuning the intensity of ER stress to achieve the best protection, whilst suppressing possible adverse effects due to toxic UPR activation. Several neuroprotective compounds and treatments function by inhibiting activation of the PERK-eIF2α-CHOP pathway in models of PD. For instance, administration of candesartan cilexetil, a selective and high-affinity Angiotensin II receptor antagonist, reduced ER stress toxicity induced by rotenone in a rat model of PD as evidenced by inhibition of ATF4, CHOP, and p53 upregulated modulator of apoptosis (PUMA) (Wu et al., 2013). Another potential therapeutic strategy is the induction of ER hormesis by hypothermia, which has been shown to protect human cortical neurons by priming proteostatic pathways including adaptive outputs of the UPR (Mollereau, 2015, Rzechorzek et al., 2015). Adaptive UPR responses in human neurons were required for hypothermic protection against both oxidative and ER stress – important contributors to acute and chronic neuronal injury (Rzechorzek et al., 2015). However, in contrast to previous work in prion-diseased mice (Moreno et al., 2012), Rzechorzek et al. showed that PERK was an important contributor to neuroprotective adaptation of the UPR and proposed that cold-shock proteins would interact with this pathway – a hypothesis recently supported for cold-inducible RNA binding motif-3 (RBM3) *in vitro* (Zhu et al., 2015).

Further evidence that UPR manipulation represents a promising therapeutic strategy specifically in PD unexpectedly came from tobacco users. Indeed, multiple epidemiological studies have shown that smokers are less prone to develop PD than non-smokers (Ritz et al., 2007). One proposed mechanism is that chronic mild activation of nicotinic receptors confers neuroprotection. Nicotine has been shown to be protective in several models of PD (Quik et al., 2015). This could arise from the upregulation of neuronal nicotinic acetylcholine receptors (nAChRs) that occurs via nicotine-mediated pharmacological receptor chaperoning within the ER (Henderson and Lester, 2015). A recent study found that the protection mediated by nicotine is due to the attenuation of the UPR in DA neurons (Srinivasan et al., 2016). Overall, the findings of many recent studies modeling PD as well as other neurodegenerative diseases reflect the complex nature of fine-tuning proteostatic mechanisms, suggesting that cell type and disease stage may significantly influence the outcome.

## 3 Adaptation for Wolfram syndrome?

Wolfram syndrome is one of the best examples that highlights the existence of a tight UPR control to avoid its dysregulation, loss of proteostasis and pathology. Wolfram syndrome is characterized not only by endocrine manifestations such as early-onset diabetes mellitus and diabetes insipidus, but also by neurological dysfunction including progressive optic nerve atrophy, ataxia beginning in early adulthood, brain stem atrophy, and psychiatric manifestations (Barrett et al., 1995, Fonseca et al., 2009, Mollereau et al., 2014, Urano, 2016). Wolfram syndrome is caused by mutations in the *WFS1* gene encoding an ER-resident membrane glycoprotein which regulates cellular calcium homeostasis (Inoue et al., 1998, Lu et al., 2014). First described in pancreatic β cells and neurons, WFS1 attenuates the UPR by specifically targeting ATF6α for degradation by the proteasome and stabilizing HRD1 (Fonseca et al., 2010). In the visual system, loss of WFSI function induces an exacerbated ER stress leading to optic nerve atrophy and impairment of visual function (Bonnet Wersinger et al., 2014, Inoue et al., 1998). Thus WFS1 is a critical regulator of the UPR, which limits its hyperactivation and neuronal cell death. In Wolfram syndrome, UPR adaptation is likely to proceed in a limited fashion because these patients develop neurodegeneration over a protracted period. It was previously shown that IRE1 inhibits ER membrane permeabilization mediated by Bax and Bak (and thus apoptosis) in cells undergoing ER stress (Kanekura et al., 2015). Inhibition of IRE1 signaling led to the accumulation of the BH3 domain-containing protein Bnip3, which in turn triggers the oligomerization of Bax and Bak in the ER membrane and ER membrane permeabilization. Consequently, in response to ER stress, cells lacking IRE1 are susceptible to the leakage of ER contents, which is associated with the accumulation of calcium in mitochondria, oxidative stress in the cytosol, and ultimately cell death. Thus increasing proteasomal flux, counteracting ER membrane permeabilization by IRE1 or priming other UPR branches to resist the downstream effects of *wsf1* mutation may prove to be valuable therapeutic approaches for Wolfram syndrome.

## 4 Adaptation to ischemia

### 4.1 Cellular responses to ischemia/reperfusion

**Fig. 2:**
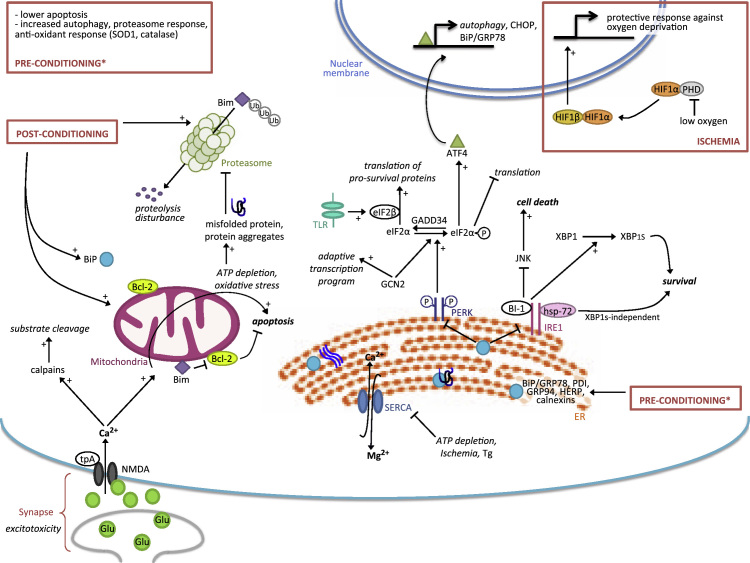
The adaptive UPR in brain ischemia. In brain ischemia, excitotoxicity is induced by an excess of glutamate which overactivates NMDA receptors. This leads to rapid influx of Ca^2+^ into the cytoplasm. The increase of intracellular Ca^2+^ has several deleterious consequences including the activation of calpains that cleave many substrates leading to proteostatic disturbance, the loss of calcium homeostasis in the mitochondria followed by oxidative stress and ATP depletion, and inhibition of the SERCA pump at the ER with subsequent UPR activation. Ischemic preconditioning induces a wide range of protective responses favoring UPR adaptive arms (BiP, HERP), autophagy, the antioxidant response (SOD1, catalase) and proteasomal activity. Postconditioning also increases proteasome activity, which degrades Bim and increases Bcl-2 hence reducing apoptosis. These treatments awaken cellular adaptive mechanisms which, together with HIF1 responses, allow the cell to better resist stress and favor a fast return to normal proteostasis after injury. Moderate stimulation of the UPR activates its adaptive pathways, which is essential to restore protein homeostasis. PERK phosphorylates eIF2α and inhibits translation which is at first beneficial because it reduces the load of misfolded proteins. GCN2 also phosphorylates eIF2α and induces an adaptive program that is independent of this phosphorylation event. The dephosporylation of eIF2α by GADD34 restores protein synthesis. This is favored by eIF2β (induced by TLR activation) and allows expression of survival proteins and progressive resumption of proteostasis. IRE1 is a key adaptive response factor in ischemia; its specific modulation by interacting with Hsp72 or BI-1 promotes XBP1-dependent and independent responses to ensure cell survival while inhibiting JNK mediated cell death. Tg: thapsigargin.

Brain ischemia is a major cause of adult mortality and disability and manifests by a reduction of blood flow to the brain, resulting in a lack of cellular oxygen, glucose, and energy, altered cellular homeostasis and ultimately cell death (Doyle et al., 2008, Hofmeijer and van Putten, 2012). Global ischemia affects the entire brain after cardiac arrest, whereas focal ischemia follows an ischemic stroke after vessel occlusion. An important event during brain ischemia is the excitotoxicity that ensues due to the release of glutamate, its extracellular accumulation and the subsequent post-synaptic toxic activation of glutamate receptors (Fig. 2, Rossi et al., 2000). This triggers a massive increase in intracellular calcium, leading to the activation of calpains (Ca^2+^-dependent proteases), which in turn cleave many cellular substrates such as proteins of the cytosolic compartment, plasma membrane, synaptic vesicles and mitochondria (Bevers and Neumar, 2008). To date, there are two main therapies to enable reperfusion: thrombolysis by tissue-type plasminogen activator (tPA) (NINDS, 1995) and more recently mechanical removal of the clot by thrombectomy (Ding, 2015). Due to technical limitations, thrombolysis by tPA remains the gold standard treatment, aiming to dissolve the blood clot and restore cerebral blood flow to ischemic tissues. However, the therapeutic window of thrombolysis is restricted to the first few hours after stroke onset and possible deleterious effects due to reperfusion (termed ischemia reperfusion injury, IRI) have to be considered. Despite a clear beneficial effect overall, in certain conditions tPA can trigger neurotoxicity (Lemarchant et al., 2012) and reperfusion induces a massive burst of reactive oxygen species and calcium overload (Bull et al., 2008, Parsons et al., 1999). The hypoxia-inducible transcription factors (HIF-1α, -2α, -3α) are essential actors in the cellular response to hypoxia. Low oxygen levels are detected by the oxygen sensors prolyl-hydroxylases (PHD), which become inactive. This in turn relieves the inhibition of PHD on HIF1α, allowing its dimerization with HIF1β and the expression of multiple genes involved in the protective response against oxygen deprivation (Semenza, 2007).

### 4.2 Ischemic tolerance

The phenomenon of ischemic preconditioning was first established in the heart, but studies from the last two decades have provided compelling evidence that it also exists in the brain (N et al., 2015, Stetler et al., 2014). Robust evidence of neuronal ischemic preconditioning has been developed in cellular, tissue culture and animal models, *i.e.* sublethal ischemic insults conferring protection against subsequent toxic ischemic insults has helped to elucidate mechanistic elements of these adaptive responses (Caldeira et al., 2014, Rybnikova and Samoilov, 2015). Using hippocampal and olfactory cortical slices, it has been demonstrated that a single short anoxia or rapid cycle of anoxia increases the resistance to severe anoxia, preventing calcium overload (Perez-Pinzon et al., 1999). Another technique of hypoxic preconditioning is exposure to mild hypobaric hypoxia that naturally occurs at moderate high altitude or experimentally in a hypobaric chamber. It induces reprogramming of cardio-pulmonary and metabolic processes, including erythropoiesis, vascular remodeling, pulmonary changes and cardiac hypertrophy (Rybnikova and Samoilov, 2015). The protection that is activated by hypoxic preconditioning involves several neuroprotective pathways that contribute to the adaptive response. For example, mild stimulation of NMDA receptors is known to induce adaptation rather than excitoxicity and has been reviewed elsewhere (Caldeira et al., 2014, Hardingham and Bading, 2010, Shpargel et al., 2008, Watters and O׳Connor, 2011). Neuroprotection also relies on cellular processes implicated in proteostasis such as the UPR, the UPS or autophagy, but also the expression of neuroprotective factors such as HIF1 and tPA.

### 4.3 UPR in ischemia

During stroke, the loss of calcium homeostasis triggers ER stress and the UPR. Functionally, ischemia inhibits SERCA - the pump that is essential for the uptake of Ca^2+^ into the ER (Fig. 2, Parsons et al., 1997). Subsequently, the loss of calcium homeostasis induces ER stress (Lehotsky et al., 2009, Paschen, 2003). Activation of the UPR has been observed in several rodent models of ischemic stroke (DeGracia and Montie, 2004, Nakka et al., 2010, Su and Li, 2015). In these models, it was shown that inhibition of protein synthesis requires PERK-dependent phosphorylation and inhibition of eIF2α. Hypoxia also stabilizes the UPR transcription factor ATF4 to promote an adaptive response (Scortegagna et al., 2014). Furthermore, IRE1 is activated during stroke, which leads to the expression of chaperones and ERAD proteins. ATF6 is more difficult to detect but was activated after middle cerebral artery occlusion (MCAO) in rats (Rissanen et al., 2006). Although protein synthesis is strongly inhibited during ischemia, a few proteins including CHOP are upregulated. CHOP was detected in several rodent models of ischemia including those involving bilateral common carotid artery occlusion, which induces global brain ischemia (Tajiri et al., 2004). Similar results were obtained in other models of brain ischemia (Nakka et al., 2010, Osada et al., 2010, Paschen et al., 1998, Paschen et al., 2003, Roberts et al., 2007). CHOP induces the expression of apoptotic genes and the translational apparatus leading to increased protein synthesis, ER protein misfolding, oxidative stress and cell death (Han et al., 2013). Moreover, mice deficient in CHOP are protected from the injury induced by bilateral common carotid artery occlusion, indicating that CHOP is a key apoptotic player during the ischemic insult (Tajiri et al., 2004).

The current dogma therefore is that the PERK branch of the UPR is protective under modest UPR activation but contributes to cell death during severe acute stress (Rutkowski et al., 2006; Walter and Ron, 2011). This dual function of PERK is reflected at the level of eIF2α phosphorylation, controlled by the phosphatase GADD34 (*growth arrest and DNA damage–inducible 34*), the regulatory subunit of protein phosphatase 1 which helps to resolve the UPR (and mRNA translation) once proteostasis is re-established. Selective inhibition of this pathway via CHOP deletion (and thus reduced GADD34 activation) can protect from ER stress by inducing a prolonged phosphorylation of eIF2α (Marciniak et al., 2004; Harding et al., 2009). However, prolonged phosphorylation of eIF2α can also lead to cell death, in particular in secretory cells as observed in pancreatic beta cells treated with salubrinal, a selective inhibitor of eIF2α phosphatases (Boyce et al., 2005; Cnop et al., 2007). It is thus clear (and intuitive) that prolonged inhibition of protein synthesis can lead to cell death (Cnop et al., 2007) and overcoming this translational repression may prove to be an important therapeutic target, as proposed for neurodegeneration (Moreno et al., 2012). Interestingly, it has been suggested that Toll-like receptors (TLRs) inhibit phosphorylation of eIF2α and CHOP expression by activating eIF2β, a guanine nucleotide exchange factor (GEF). This maintains a long lasting physiological ER stress, enabling the synthesis of essential proteins in macrophages whilst benefiting from the protective arms of the UPR (Woo et al., 2012).

### 4.4 UPR in ischemic tolerance

Despite several lines of evidence that ER stress and UPR activation promote cell death in ischemia models, other studies have shown that the UPR is also activated by transient preconditioning treatments and contributes to neuroprotection. One study examined the temporal expression of chaperones and folding proteins in rats submitted to transient global ischemia, using the 2-vessel occlusion model. The authors found that while heat shock protein 70 (Hsp70) is first expressed in the cytoplasm (within 30 min), this is followed by the induction of Hsp60 in the mitochondria, and then HERP, GRP78, GRP94, calnexin and PDI in the ER lumen at a later stage (4–24 h) (Truettner et al., 2009). Another study in *C. elegans* showed that the UPR is required for resistance to hypoxia in animals carrying a mutation in the *rrt-1* gene*,* encoding an arginyl-transfer RNA (tRNA) synthetase. This enzyme is essential for protein translation and the level of hypoxia resistance in *C. elegans* was inversely correlated to the translation rate (Anderson et al., 2009). In a follow-up study, the same group found that resistance to hypoxia required IRE-1 but not XBP-1 or ATF6. In addition GCN2, a kinase known to phosphorylate eIF2α upon amino acid deficiency, induces an adaptive transcriptional response required for adaptation to hypoxia (Mao and Crowder, 2010). Interestingly the phosphorylation of eIF2α by GCN2 was not required for this adaptation, suggesting that this mechanism is independent of translational suppression. In another *C. elegans* study, it was proposed that the Heterochromatin Protein 1 (HP1) homolog HPL-2 plays an important role in the induction of UPR during preconditioning (Kozlowski et al., 2014). Loss of HPL-2 in animals led to a protective response dependent on XBP1. Although resistance of these animals to hypoxia was not tested in this study, these results suggest that chromatin structure may be modulated by stress to induce UPR-mediated protection, either through direct transcriptional effects or through more global changes in chromatin organization.

The protective pathways activated by preconditioning downstream of the UPR are yet to be fully elucidated. One possibility is that the adaptive response curtails the ER stress-induced cell death that might otherwise occur in response to ischemic injury. This idea was recently supported by a study exploiting the brain protection mediated by postconditioning i.e. a conditioning treatment applied shortly after injury (Liu et al., 2014). The authors found that postconditioning of rats previously subjected to ischemia/reperfusion increased the protein levels of chaperone BiP/GRP78 and the anti-apoptotic factor Bcl-2 but decreased phosphorylated-eIF2α, and the expression of pro-apoptotic CHOP, caspase-12, Bcl-2-interacting mediator of cell death (Bim) and cleaved-caspase-3 (Liu et al., 2014). The molecular switch that favors pro-survival in ischemia is still unknown. A potential candidate for regulating this switch is Hsp72, which has been shown to reduce tissue injury in experimental models of stroke and myocardial ischemia (Morimoto et al., 1997). Indeed, it was proposed that Hsp72, which is induced by ER stress, enhances survival by interacting with and activating IRE1 endoribonuclease activity. This results in XBP1 splicing, activation of its target genes and the attenuation of apoptosis in ER stress conditions (Gupta et al., 2010). Another possible candidate to regulate the switch toward cell death or survival is Bax-inhibitor-1 (BI-1) (Chae et al., 2003, Chae et al., 2004). In models of hepatic and liver ischemia-reperfusion, BI-1 was shown to protect cells from extensive ER stress (Bailly-Maitre et al., 2006). In contrast to Hsp72, which increases IRE1 activity, BI-1 limits IRE1 endoribonuclease activity (Lisbona et al., 2009). Thus BI-1 may protect cells by suppressing IRE1 signaling. Interestingly the lack of BI-1 also resulted in neuroprotection under nutrient deprivation. In BI-1 deficient cells, an increased IRE1 led to JNK activation and autophagy (Castillo et al., 2011). BI-1 is also an important regulator of neuronal survival *in vivo* during ischemia-reperfusion (Krajewska et al., 2011). BI-1-deficient mice display increased sensitivity to cerebral ischemia-reperfusion injury by MCAO. Reversibly, enforced neuronal expression of BI-1 confers protection from IRI in brain. Reduced phosphorylation of the JNK substrate c-JUN was observed in brain tissue after MCAO, consistent with the notion that BI-1 affords neuroprotection by suppressing IRE1 signaling (Krajewska et al., 2011). Thus BI-1 can favor death or survival upon ER stress activation and further work is needed to understand how this dichotomy might be exploited for therapeutic gain.

Intriguingly, studies implicate a major requirement for oxidative stress in ER stress-induced cell death. Either deletion of CHOP or providing antioxidants prevented cell death when challenged with protein misfolding in the ER (Back et al., 2009, Han et al., 2015, Malhotra and Kaufman, 2007, Malhotra et al., 2008, Song et al., 2008). Surprisingly, antioxidant treatment also improved protein folding in the ER, indicating an intimate connection between protein misfolding and oxidative stress (Malhotra et al., 2008). More recent studies indicate that protein misfolding in the ER decreases activity of complex I of the respiratory complex, leading to oxidative stress.

### 4.5 Autophagy in ischemic preconditioning

Autophagy is an important protective mechanism that is induced by ischemic preconditioning (Carloni et al., 2008, Sheng and Qin, 2015). Preconditioning treatment reduced cell damage induced by oxygen and glucose deprivation (OGD) in cultured cortical neurons, whereas inhibition of autophagy by 3-MA or bafilomycin A1, increased caspase-12, caspase-3 and CHOP protein levels and suppressed the neuroprotection induced by preconditioning (Sheng et al., 2012). Importantly, inhibition of ER stress by salubrinal restored neuroprotection mediated by preconditioning in the presence of 3-MA. Moreover, preconditioning with ER stressors prior to transient MCAO in mice and OGD in neurons has been shown to afford neuroprotection through eIF2/ATF4-dependent Parkin-mediated induction of mitophagy (Zhang et al., 2014). Thus, in the context of ischemia-reperfusion, there is a hormetic proteostatic mechanism that connects ER stress and autophagy whereby ER stress-induced apoptosis is inhibited. It seems that the eIF2/ATF4 pathway plays a pivotal role in ischemic preconditioning; on one hand by compensating for autophagy overload through global translational suppression, and on the other by promoting clearance of damaged mitochondria though mitophagy. In addition, inhibition of ER stress by autophagy was observed after neonatal hypoxia/ischemia (Carloni et al., 2014). Overall autophagy is an important proteostatic response that protects neurons after preconditioning, and this protective effect can be modulated by manipulating the UPR. These studies highlight the elegant coordination of multiple proteostatic mechanisms in neuronal preconditioning and that upregulation of a proximal defense strategy (autophagy) can reduce the need for a downstream rescue (the UPR). The cyclical crosstalk and context-dependent redundancy between these pathways means that proteostatic disturbances can be effectively ‘triaged’ under conditions of metabolic compromise in the healthy cell. This provides a network of targets that might be adjusted with synergetic benefit in both acute and chronic neuronal injury.

### 4.6 UPS in ischemia

It is thought that the UPS (*Box 2*) has an important role during ischemia. Indeed, several *in vivo* studies have reported that ischemia depletes free ubiquitin and leads to the accumulation of ubiquitinated proteins that tend to form aggregates in neurons (Hu et al., 2000, Hu et al., 2001). For example, in a global ischemia model induced by a transient two-vessel occlusion in rats, neurons in the hippocampal CA1 region showed accumulation of ubiquitin-conjugates in aggregate-like clusters (Hu et al., 2000). These clusters were preferentially found in dying neurons suggesting that proteasomal dysfunction could be the cause of neuron death (Yamashiro et al., 2007). It seems therefore that ischemia is associated with a perturbation of proteostasis; an accumulation of Ub-proteins into aggregates that may interfere with optimal UPS function. The impairment of proteasomal function by pathological protein aggregates was also observed in neurodegenerative diseases (Ciechanover and Kwon, 2015). For example, in prion disease the infectious form of the prion protein (PrP-Sc) with its exposed beta sheet composition, interacts and interferes with the gate opening of the proteasome, limiting the entry of substrates (Andre and Tabrizi, 2012). This is reminiscent of AD, in which ubiquitinated and oligomeric tau protein interacts with and inhibits the recognition site of the 19S proteasome subunit (Tai et al., 2012). In addition, the proteolytic core of the proteasome can become blocked by hyperphoshorylated tau leading to ERAD impairment and thereafter UPR activation (Keck et al., 2003; Abisambra et al., 2013). Whether this represents an adaptive or maladaptive response remains controversial. Indeed, since mild UPR activation can precondition human neurons, rapid tau hyperphosphorylation under hypothermic conditions has been proposed as a trigger for UPR-mediated proteostatic priming in response to cooling (Rzechorzek et al., 2015, Rzechorzek et al., 2016).

Whether the formation of aggregates containing ubiquitinated proteins or simply monomeric and oligomeric misfolded proteins induce toxicity during ischemia is unclear and is hotly debated in neurodegenerative disease. It was proposed that protein aggregates trap translational components, chaperones and protein folding enzymes and thus contribute to proteostatic disruption in brain ischemia (DeGracia and Montie, 2004, Liu et al., 2005). However, a more recent study showed that reperfusion rather than ischemia leads to the formation of aggregates after transient MCAO. This might be due to massive oxidation of proteins caused by the burst of reactive oxygen species (ROS) (Grune et al., 2004) and echoes the adverse effects of the re-warming phase after therapeutic cooling for brain ischemia (Choi et al., 2012, Rzechorzek et al., 2016). Furthermore, permanent ischemia did not lead to aggregate formation despite maximally impairing the proteasome (Hochrainer et al., 2012). This study rather proposes an alternative mechanism in which the impairment of the proteasome is due to the selective processing of the 26S proteasome subunit Rpn10 by calpain (Huang et al., 2013). Thus, aggregates of ubiquitinated proteins may not be the relevant mechanism for proteasome impairment and neuronal death. This is further supported by an elegant study with live imaging tracking of neurons expressing Huntingtin-polyQ proteins showing that aggregates (inclusion bodies) are preferentially observed in surviving neurons – i.e. neurons that do not accumulate aggregates tend to die more rapidly (Arrasate et al., 2004). These results indicate that aggregates are protective and may function as a sink by trapping toxic monomeric or oligomeric Htt-polyQ proteins (Arrasate and Finkbeiner, 2012). Thus, impairment of proteasome function in HD and also in brain ischemia may be due to the accumulation of relatively soluble toxic monomers and oligomers, rather than insoluble protein aggregates. Conceptually, we can consider the following scenario during the course of disease: in the early phase, small amounts of modified/misfolded protein and UPS impairment would promote UPR and ERAD – and potentially hormesis if the insult is mild. Long-term, or if the insult is too acute or severe, CHOP-mediated apoptosis or regulated necrosis is expected.

### 4.7 UPS in ischemic tolerance

The UPS has an important role in the adaptive response to ischemia. First, it was shown that activation of the UPS during ischemic tolerance induces the ubiquitination and degradation of pro-apoptotic factors. This is the case for Bim which is ubiquitinated and targeted to proteasome-mediated degradation through preconditioning induced by transient OGD (30 min). This resulted in the protection of cultured cortical neurons submitted to a more prolonged OGD (120 min) (Meller et al., 2006). Proteasome-dependent degradation of Bim and neuroprotection was also observed by preconditioning of adenosine A1 receptors with adenosine in cultured rat cortical neurons (Ordonez et al., 2010). This led to an increased resistance to apoptosis – a phenomenon also observed with the upregulation of Bcl-2 proteins by H_2_O_2_-mediated oxidative preconditioning (Calabrese, 2016b). In addition, it was shown that during rapid ischemic preconditioning the UPS protects neurons from excitotoxicity via post-synaptic remodeling (Meller et al., 2008). This is consistent with an immediate and active role of the UPS in ischemic tolerance.

An important perspective for logistically-feasible application of neuroprotective conditioning in the clinic is that treatment may also be of value after the injury has occurred (postconditioning). Indeed, conditioning has exhibited a protective effect even if administered a few hours after trauma (Stetler et al., 2014). For example, it was shown that ischemic postconditioning cycles reduced the size of cerebral infarction induced by MCAO in adult rats. The postconditioning treatment improved brain integrity, which was associated with an increased activity of the proteasome and anti-oxidant (SOD, catalase) enzymes with a subsequent reduction of oxidized proteins and aggregates (Li et al., 2012). Postconditioning-induced neuroprotection was also associated with restoration of proteasome function in neurons of the hippocampal CA1 region in rats subjected to the transient two-vessel-occlusion model of global ischemia (Liang et al., 2012). These studies and others indicate that a functional UPS is essential for ischemic tolerance and that proteasome inhibition can lead to neuronal death. This hypothesis is supported by the results showing that IU1, an inhibitor of Usp14 (a deubiquitinase that acts as a negative regulator of the proteasome), reduces the infarct volume resulting from transient MCAO in mice. The effects of IU1 were correlated with regulation of REST, a protein whose expression is increased in neurons destined to die in brain ischemia (Doeppner et al., 2013). In these studies IU1 was administered before the ischemic injury and, therefore, future studies should characterize the effect of proteasome activation after the lesion has been induced.

Despite the results above showing a role for proteasome inhibition in neuronal demise in brain ischemia, it is interesting to note that proteasome inhibitors have induced neuroprotection in several models of stroke. This apparent contradiction may be explained by the effect of proteasome inhibitors on the suppression of the immunoproteasome and inflammation that occurs after stroke. Indeed, proteasome inhibitors such as MLN519 reduce inflammation by downregulating NF-κB and downstream inflammatory genes and by decreasing the recruitment of inflammatory cells into the brain (Berti et al., 2003, Phillips et al., 2000, Williams et al., 2003, Williams et al., 2004, Williams et al., 2005; Zhang et al. 2001). Another proteasome inhibitor, BSc2118, protected from stroke in mice subjected to intraluminal MCAO, by stabilizing the blood–brain barrier and upregulating HIF1-α (Doeppner et al., 2012). A more recent study by the same group showed that a single intraperitoneal injection of BSc2118 induced a sustained brain recovery by acting on the peripheral immune response and inhibiting the immunosuppression that is associated with stroke (Doeppner et al., 2015).

### 4.8 tPA in ischemic tolerance

tPA is not only a drug injected in the acute phase of cerebral ischemia to restore the blood flow. It is also a serine protease synthetized and released by neurons with many, and sometimes opposite, effects in the brain (Chevilley et al., 2015). Due to its proteolytic activity, tPA cleaves many cerebral substrates which induce important cell fate or adaptive mechanisms. During development and axon growth, tPA activates plasminogen into plasmin to degrade the extracellular matrix (Garcia-Rocha et al., 1994). Many other tPA substrates have been identified, including the GluN1 subunit of NMDA receptors (Nicole et al., 2001). tPA also has a wide range of other functions including activation of ADAMTS-4 to promote neuroplasticity (Lemarchant et al., 2014), conversion of pro-BDNF into BDNF to promote LTP in the hippocampus (Pang et al., 2004), and activation of PDGF-C (Fredriksson et al., 2004) to promote cell proliferation.

## 5 Adaptation in glioblastoma

Cancer cells often activate adaptive responses to cope with oncogenic and environmental stresses. As such, cancer cells have not only to deal with an accelerated metabolism that can be caused by oncogene overexpression (*i.e.* MYC) but also with a challenging microenvironment (i.e. nutrient starvation or hypoxia). High-grade glioma (also known as glioblastoma multiforme, GBM) is the most frequent and aggressive brain cancer, which still lacks effective therapeutics. It is associated with a strong UPR-mediated adaptive response (Pierre-Jean Le Reste et al., see associated manuscript of this series). GBM is notoriously resistant to treatment and recurrence leads to a poor clinical outcome (Louis et al., 2007, Pyrko et al., 2007). The UPR has become a therapeutic target of interest in cancer and one potential strategy is to either genetically or pharmacologically invalidate UPR components to reduce cancer cell resistance to their environment and to increase their sensitivity to treatment (Hetz et al., 2013, Mollereau, 2013). This has been illustrated with BiP/GRP78, which is frequently overexpressed in cancer including GBM (Martin et al., 2013, Prabhu et al., 2012, Pyrko et al., 2007). In addition to BiP, the three branches of the UPR have also been involved in the control of GBM characteristics. Indeed IRE1, which is the fifth most commonly mutated kinase in human cancer, contributes to the development of GBM in experimental models (Auf et al., 2010, Chevet et al., 2015, Dejeans et al., 2012, Drogat et al., 2007, Jabouille et al., 2015, Pluquet et al., 2013). More precisely, IRE1 signaling pathways were shown to impact on GBM tumor angiogenesis through the regulation of proangiogenic and proinflammatory chemokines (Auf et al., 2010, Pluquet et al., 2013). Moreover, regulated IRE1 dependent decay of mRNA (RIDD) activity was shown to significantly contribute to GBM infiltration through the degradation of SPARC mRNA (Dejeans et al., 2012). In a recent study, mutants for both the IRE1 kinase and endoribonuclease were used to determine the specific contribution of each activity. It was shown that while the RNAse activity of IRE1 is dispensable for neovascularization, the inhibition of RNAse resulted in increased glioma motility (Jabouille et al., 2015). Finally, IRE1 activates the epidermal growth factor receptor (EGFR) pathway, often found deregulated in GBM, by upregulating its ligand epirgulin. This is independent of IRE1 kinase activity and of XBP1, but instead requires JNK activation by IRE1 (Auf et al., 2013). Finally, in human GBM samples high levels of XBP1 splicing correlated with a poorer prognosis (Pluquet et al., 2013). These data collectively point toward the seminal role of IRE1 in the development and progression of GBM. More recently the PERK and ATF6 arms of the UPR were also shown to be involved in the control of GBM development. Indeed, the PERK pathway was implicated in the regulation of GBM cell metabolism (Hou et al., 2015) and response to treatment (Hamed et al., 2010, Yacoub et al., 2010), whereas the ATF6 pathway was recently reported to contribute to GBM resistance to radiotherapy (Dadey et al., 2015). Interestingly, a high resolution CRISPR screen also indicated the contribution of the ATF6 arm of the UPR to GBM development (Hart et al., 2015). In summary, these results demonstrate the essential role of UPR signaling pathways in GBM biology, and indicate their potential therapeutic relevance.

## 6 Conclusions

Cellular adaptation to stress involves the activation of multiple protective pathways that contribute to restore proteostasis. In neurodegenerative diseases such as PD, adaptive mechanisms that include the UPR, the UPS, autophagy and the anti-oxidant responses allow neurons to cope with the accumulation of misfolded proteins for decades. In recent years, researchers have identified the molecular factors and compounds that regulate these adaptive responses. In particular, pre- or postconditioning strategies that elicit a mild insult and promote adaptive UPR responses seem particularly promising to treat acute brain injury such as ischemia. These approaches have also generated interesting results in animal models of PD which, in contrast to brain ischemia, progresses slowly over the lifetime of the individual (Mollereau, 2015). UPR preconditioning may thus be relevant to human patients with neurodegenerative disease. Unexpectedly, epidemiologic studies revealed that smokers have a lower incidence of PD than non-smokers (Ritz et al., 2007). Almost pure nicotine, which can be delivered from patches or e-cigarettes, can prime the UPR at a low level, hence favoring its adaptive protective response (rather than its maladaptive outputs) (Srinivasan et al., 2016). The results from a large clinical trial, testing the neuroprotective effects of a transdermal nicotine patch in early PD, are awaited (https://clinicaltrials.gov/show/NCT01560754). Convergence of research efforts to illuminate the proteostatic pathways dictating cell fate will accelerate the discovery of ‘pleiotropic targets’ – targets that can be manipulated to impede cellular survival mechanisms where they are unwanted (i.e. tumor growth) and promote these mechanisms where they are failing (neurodegeneration).

## Box 1 the unfolded protein response

The UPR is an adaptive cellular response that aims to safeguard proteostasis in the face of misfolded protein accumulation within the endoplasmic reticulum (ER) (Walter and Ron, 2011, Wang and Kaufman, 2016). Under basal conditions, the UPR chaperone binding immunoglobulin protein (BiP/GRP78) associates with (and thereby inhibits) the three main ER resident transmembrane stress tranducers: protein kinase RNA-like ER kinase (PERK), inositol-requiring enzyme 1 (IRE1), and activating transcription factor 6 (ATF6). When ER stress is detected, BiP/GRP78 associates with misfolded protein, thus releasing these three ER stress signal transducers, which in turn activate a tripartite signaling cascade to restore proteostasis. Downstream UPR outputs include attenuation of protein synthesis, increased expression of chaperones that assist with protein folding, and degradation of misfolded proteins via ER-associated protein degradation (ERAD) (Hetz et al., 2015). UPR activation also increases proteasomal activity and macroautophagy (hereinafter referred to as autophagy) (Klionsky et al., 2016). Activated PERK phosphorylates the alpha subunit of eukaryotic translation initiation factor 2α (eIF2α), resulting in the rapid and transient attenuation of protein translation and the reduced import of newly synthesized proteins into the ER. Despite the downregulated translation of most transcripts, translation of activating transcription factor 4 (ATF4) mRNA is induced; this transcription factor then facilitates the expression of ER resident chaperones as well as proteins involved in autophagy, anti-oxidant responses, amino acid metabolism and (in the case of insurmountable ER stress) C/EBP-homologous protein (CHOP) which induces apoptosis (Walter and Ron, 2011). ATF4 and CHOP can also form heterodimers that activate genes encoding ER adaptive functions as well as increasing the translational machinery to restore protein synthesis (Han et al., 2013). The second and most conserved proximal UPR transducer, IRE1, carries both Ser/Thr kinase and endoribonuclease activities within its cytoplasmic domain. Upon dimerization and autophosphorylation, IRE1 endoribonuclease initiates the unconventional splicing of x-box binding protein 1 (XBP1) mRNA, producing the transcription factor XBP1s (spliced XBP1). XBP1s in turn induces the expression of genes encoding proteins involved in co-translational translocation, lipid synthesis, inflammation and other processes, as well as signal recognition particle receptor, ER chaperones, and ERAD proteins (Hetz et al., 2011, Sidhu et al., 2015). IRE1 endoribonuclease also degrades specific mRNAs associated with the ER, through a mechanism known as regulated IRE1-dependent decay (RIDD) (Coelho and Domingos, 2014, Maurel et al., 2014). Finally, IRE1 also activates the Jun amino terminal kinase (JNK) stress pathway and the apoptosis signal regulating kinase 1 (ASK1) (Urano et al., 2000). The third UPR transducer, ATF6, translocates to the Golgi where it is cleaved by site 1 and site 2 proteases (S1P and S2P). The released cytosolic ATF6f is a transcription factor, which induces the expression of XBP1 and ERAD proteins (Walter and Ron, 2011). ATF6α is the major transcription factor that activates genes encoding the adaptive UPR (Wu et al., 2007). Considering the major role of the UPR in proteostasis, it is intuitive that deregulation of these processes are pathogenic (Wang and Kaufman, 2012)

## Box 2 the ubiquitin proteasome system

The first barrier of defense against the accumulation of misfolded proteins is the ubiquitin (Ub)-proteasome system (UPS) (Ciechanover, 2015). In this selective proteolytic system, Ub is first activated by Ub-activating enzyme E1 and transferred to Ub-conjugating enzyme E2. The Ub is then transferred to a protein substrate via a specific Ub ligase E3, which varies depending on its substrate. Several Ubs are successively added to the protein leading to a chain of four or more Ub. Ub-conjugated proteins are delivered to the 26S proteasome, a cylindrical structure that is composed of a catalytic 20S subunit and one or two 19S regulatory particles (Clague and Urbe, 2010; Finley, 2009). Proteins are deubiquitinated, unfolded and cleaved into small peptides by peptidylglutamyl-like (cleaving after acidic residues), trypsin-like (cleaving after basic residues) and chymotrypsin-like (cleaving after hydrophobic residues) activities, while going through the proteasome machinery (Tanahashi et al., 2000). Dysfunction of the UPS due to mutations in the ubiquitinase/deubiquitinase and proteasome machineries help us to understand the importance of the UPS to proteostasis. Indeed, UPS dysfunction is a common hallmark of protein misfolding disorders including neurodegenerative diseases such as AD, HD, PD, ALS, prion diseases and ischemia (Morimoto, 2008, Rubinsztein, 2006 Caldeira et al., 2014, Ciechanover and Kwon, 2015, Dennissen et al., 2012)
